# Invader removal triggers competitive release in a threatened avian predator

**DOI:** 10.1073/pnas.2102859118

**Published:** 2021-07-19

**Authors:** J. David Wiens, Katie M. Dugger, J. Mark Higley, Damon B. Lesmeister, Alan B. Franklin, Keith A. Hamm, Gary C. White, Krista E. Dilione, David C. Simon, Robin R. Bown, Peter C. Carlson, Charles B. Yackulic, James D. Nichols, James E. Hines, Raymond J. Davis, David W. Lamphear, Christopher McCafferty, Trent L. McDonald, Stan G. Sovern

**Affiliations:** ^a^US Geological Survey, Forest and Rangeland Ecosystem Science Center, Corvallis, OR 97330;; ^b^US Geological Survey, Oregon Cooperative Fish and Wildlife Research Unit, Department of Fisheries and Wildlife, Oregon State University, Corvallis, OR 97331;; ^c^Hoopa Tribal Forestry, Hoopa, CA 95546;; ^d^US Department of Agriculture Forest Service, Pacific Northwest Research Station, Corvallis, OR 97331;; ^e^US Department of Agriculture Wildlife Services, National Wildlife Research Center, Fort Collins, CO 80521;; ^f^Green Diamond Resource Company, Korbel, CA 95550;; ^g^Department of Fish, Wildlife, and Conservation Biology, Colorado State University, Fort Collins, CO 80523;; ^h^US Fish and Wildlife Service, Oregon State Office, Portland, OR 97266;; ^i^US Geological Survey, Southwest Biological Science Center, Flagstaff, AZ 86001;; ^j^US Geological Survey, Patuxent Wildlife Research Center, Laurel, MD 20708;; ^k^McDonald Data Sciences, LLC, Laramie, WY 82070;; ^l^Department of Fisheries and Wildlife, Oregon State University, Corvallis, OR 97331

**Keywords:** invasive species, removal experiment, population dynamics, competition, *Strix* owls

## Abstract

Changes in the distribution and abundance of invasive species can have far-reaching ecological consequences. Programs to control invaders are common but gauging the effectiveness of such programs using carefully controlled, large-scale field experiments is rare, especially at higher trophic levels. Experimental manipulations coupled with long-term demographic monitoring can reveal the mechanistic underpinnings of interspecific competition among apex predators and suggest mitigation options for invasive species. We used a large-scale before–after control–impact removal experiment to investigate the effects of an invasive competitor, the barred owl (*Strix varia*), on the population dynamics of an iconic old-forest native species, the northern spotted owl (*Strix occidentalis caurina*). Removal of barred owls had a strong, positive effect on survival of sympatric spotted owls and a weaker but positive effect on spotted owl dispersal and recruitment. After removals, the estimated mean annual rate of population change for spotted owls stabilized in areas with removals (0.2% decline per year), but continued to decline sharply in areas without removals (12.1% decline per year). The results demonstrated that the most substantial changes in population dynamics of northern spotted owls over the past two decades were associated with the invasion, population expansion, and subsequent removal of barred owls. Our study provides experimental evidence of the demographic consequences of competitive release, where a threatened avian predator was freed from restrictions imposed on its population dynamics with the removal of a competitively dominant invasive species.

## Significance

Invasive species can cause extinctions of native species and widespread biodiversity loss. Invader removal is a common management response, but the use of long-term field experiments to characterize effectiveness of removals in benefitting impacted native species is rare. We used a large-scale removal experiment to investigate the demographic response of a threatened native species, the northern spotted owl, to removal of an invasive competitor species, the barred owl. Removal of barred owls had a strong, positive effect on survival of spotted owls, which arrested long-term population declines of spotted owls. The results demonstrate that the long-term persistence of spotted owls will depend heavily on reducing the negative impacts of barred owls while simultaneously addressing other threats, such as habitat loss.

Invasions by nonindigenous species are a pervasive cause of global biodiversity loss (1–3). The legacies of biological invasions, such as species extinctions, can permanently alter ecosystems and have long-term consequences for the management of natural resources. Removal of invasive species has become an increasingly common response in ecological restoration programs focused on maintaining native wildlife and biodiversity (4, 5). Removal efforts that reduce invader densities may have beneficial effects to natives, but whether such efforts can stabilize or reverse declining population trends of affected species remains largely untested, especially at higher trophic levels. A detailed understanding of how control measures affect populations of terrestrial predators, for example, requires field experiments conducted at large spatial scales under a range of environmental conditions. Experimental manipulation of terrestrial predators at broad spatial scales is logistically, financially, and ethically problematic. Consequently, studies that focus on competitive interactions at higher trophic levels are often limited to short-term, observational designs that lack detailed demographic data, control populations, or sufficient spatial replication to capture species-level responses.

**Fig. 1.**
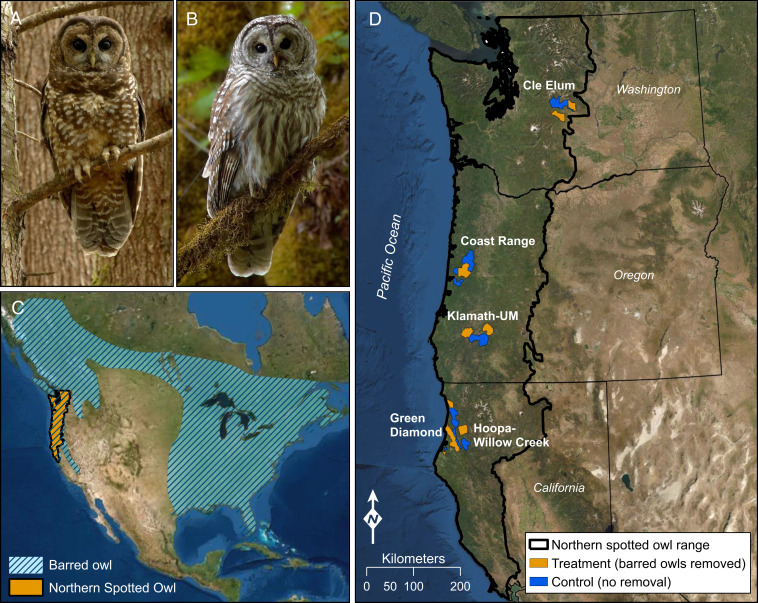
Before–after control–impact experiment used to estimate the demographic response of threatened northern spotted owls to removal of invasive barred owls. (*A*) Adult northern spotted owl (*S. occidentalis caurina*) and (*B*) adult barred owl (*S. varia*). Owl images credit: Patrick Kolar (photographer). (*C*) Overlap between the geographic ranges of northern spotted owls (US range in orange) and barred owls (hatched-blue) in North America. Ranges were approximated from https://birdsoftheworld.org/bow. Barred owls were historically limited to eastern North America. (*D*) Locations of treatment (barred owls removed) and control (no barred owls removed) segments of five long-term experimental study areas within the range of the northern spotted owl in Washington, Oregon, and California.

The conservation and management of northern spotted owls (*Strix occidentalis caurina*) is one of the largest and most visible wildlife conservation issues in United States history (6–8). The northern spotted owl, an old conifer forest obligate, was listed in 1990 as a federally threatened subspecies because of rapid declines in old-forest habitats (9). Despite over 30 y of protection under the Federal Endangered Species Act, populations have continued to decline and, in some cases, those declines have accelerated (10, 11). Long-term demographic monitoring of spotted owl populations across the species’ range identified rapid increases in the occurrence of nonnative barred owls (*Strix varia*) as a primary reason for those declines, especially in recent years (10–12). As a species native to eastern North America, barred owls began expanding their populations westward in the early 1900s. The subsequent barred owl invasion into western North America has been well documented, and the newly extended range of this species now completely overlaps that of the northern spotted owl (13, 14) (Fig. 1*C*). While congeneric barred owls are morphologically (Fig. 1 *A* and *B*) and ecologically similar to spotted owls, barred owls are larger, use smaller home ranges, and have a much broader (generalist) diet that includes many small mammal prey important to spotted owls (15, 16). Barred owls are also competitively dominant to spotted owls during territorial confrontations, and where the two species co-occur, they exhibit a high degree of overlap in patterns of habitat use (16). This combination of exploitation and interference competition, coupled with rapidly increasing numbers of barred owls in older forests throughout the Pacific Northwest, exacerbated spotted owl population declines historically triggered by habitat loss (7, 10, 17).

Mounting concerns about the threat of barred owls prompted consideration of several potential research and management options (15, 18). Among these, removal experiments were identified as having the greatest value in determining the role of barred owls in population declines of spotted owls, plus the experiments would provide a means of directly testing the effectiveness of removals as a possible management tool for spotted owl recovery (19, 20). A review of possible study designs concluded that a paired before–after control–impact (BACI) experimental design could provide the strongest inference and greatest statistical power in addressing both research and federal regulatory agency needs (20, 21). As barred owl populations continued to expand throughout the spotted owl’s range, a pilot removal experiment was initiated near the barred owl’s invasion front into California (22, 23). The study concluded that removal of barred owls, when coupled with conservation of suitable forest conditions, can slow or even reverse population declines of spotted owls. Yet, this pilot study was conducted at a time and location where barred owl populations were relatively sparse compared to spotted owls (10). Meanwhile, in Oregon and Washington, populations of barred owls had grown so rapidly that they greatly outnumbered spotted owls in many areas and were having considerable impacts on spotted owl territory occupancy (10, 17, 24), resource use (16) and, ultimately, population trends (10, 12). It was unknown whether the positive results of barred owl removal documented previously in California could be achieved in areas with different forest conditions, greater densities of barred owls, and fewer remaining spotted owls.

We used a large-scale BACI removal experiment to investigate the impact of an invasive avian predator, the barred owl, on the population dynamics of an iconic old-forest native species, the northern spotted owl. Our goal was to test the research hypothesis that trends in vital rates (survival, dispersal, recruitment) and population rates of change (λ) of northern spotted owls would be positively influenced by barred owl removal. The removal experiment was spatially replicated across five study areas with long-term demographic data on northern spotted owls (Fig. 1*D* and Table 1), where ecological factors affecting populations, including the presence of barred owls, were well documented (10–12, 25). The removal experiment capitalized on this wealth of information, which allowed us to formally assess the impacts of an invasive avian predator on the population dynamics of a closely related native predator, as well as to suggest possible mitigation measures. Our approach to examining the effect of barred owl removal on spotted owl populations was twofold. We first used long-term mark–recapture data from each study area to examine the effect of barred owl removal on annual survival and dispersal of resident spotted owls in each study area separately. We then combined data from all five study areas in a single meta-analysis of apparent survival, recruitment, and annual rate of population change (*SI Appendix*, *Supplementary Text*). The meta-analysis treated each individual study area as a unit of replication, thereby providing the strongest possible inferences on the demographic consequences of competition.

**Table 1.** Study areas and samples of color-banded owls used to estimate the effect of barred owl removal on vital rates of northern spotted owls in Washington, Oregon, and California

Study area (study area acronym)	Area (km^2^)	Total nonjuvenile spotted owls banded since 2002 (M, F)	Total barred owls removed*
Cle Elum, WA (CLE)			
Control	670	39 (22, 17)	
Treatment	604	42 (24, 18)	463
Coast Range, OR (COA)			
Control	1,015	148 (76, 72)	
Treatment	582	83 (43, 40)	1,006
Klamath-Union/Myrtle, OR (KLA-UM)			
Control	698	212 (115, 97)	
Treatment	783	198 (113, 85)	522
Hoopa-Willow Creek, CA (HUP-WC)			
Control	294	146 (70, 76)	
Treatment	348	156 (85, 71)	399
Green Diamond, CA (GDR)			
Control	727	120 (65, 55)	
Treatment	828	340 (178, 162)	95
All study areas combined			
Control	3,404	665 (348, 317)	
Treatment	3,145	819 (443, 376)	2,485

All study areas used 2002 as the start year for inclusion of demographic monitoring data.

* The period of barred owl removal for each study areas was: 2015 to 2019 (CLE), 2015 to 2019 (COA), 2016 to 2019 (KLA-UM), 2013 to 2019 (HUP-WC), and 2009 to 2014 (GDR).

## Results

### Barred Owl Removal.

We used barred owl-specific surveys to locate and monitor barred owls (26, 27). We surveyed barred owls across treatment and control areas and throughout the removal period. Removals occurred on treatment areas for 3 to 6 y during 2009 to 2019, depending on the study area (Table 1 and *SI Appendix*, Fig. S1). Barred owls detected in treatment areas were removed using 12-gauge shotguns and well-established field protocols (20, 22, 23). A total of 2,485 barred owls were removed from treatment segments of five different study areas during the experiment (Table 1). The mean number of barred owls removed per year was highly variable among study areas, ranging from a low of 15.8 barred owls per year in Green Diamond (GDR), to a high of 251.5 barred owls per year in the Oregon Coast Range (COA) (*SI Appendix*, Fig. S1).

### Survival and Movement on Individual Study Areas.

**Fig. 2.**
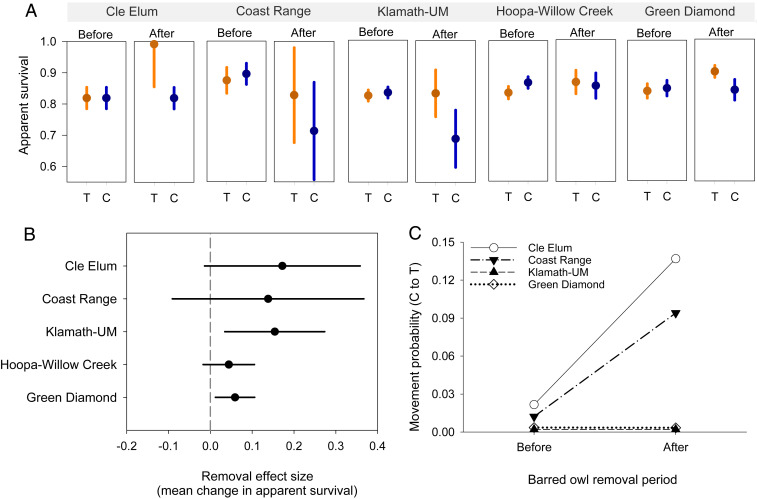
Estimated effects of barred owl removal on survival and dispersal movements of northern spotted owls in each of five individual study areas in Washington, Oregon, and California. (*A*) Weighted mean estimates of apparent survival of northern spotted owls before and after barred owls were removed. Mean survival was estimated separately for treatment (T; orange) and control (C; blue) areas in each time period using the best multistate model that included an effect of barred owl removal; estimates were weighted by the inverse of the variance of annual estimates. (*B*) Mean change in apparent survival attributable to barred owl removal in each study area, calculated using Eq. 2 with estimates shown in *A* as the mean difference in survival between control and treatment areas before and after removals. Error bars in *A* and *B* are 95% CI. (*C*) Movement probability of resident northern spotted owls from territories in the control area to territories in the treatment (removal) area before versus after removals occurred. No movement was detected on the HUP-WC study area (i.e., movement probability = 0).

We used multistate mark–recapture analysis (28–30) with 7,665 captures and recaptures of 1,721 nonjuvenile spotted owls to estimate the effect of barred owl removal on apparent annual survival and dispersal movements of spotted owls between areas with (treatment) and without (control) removal. We tested for an effect of barred owl removal on apparent survival of spotted owls by introducing a time (before–after) × treatment (control–impact) interaction to the best mark–recapture models characterizing baseline variation in sex, time, and preremoval differences between treatment and control areas (*SI Appendix*, *Supplementary Text*). There were negligible differences between treatment and control areas in apparent survival before removals (Fig. 2*A*). After removals, we observed higher estimates of apparent survival on treatment areas relative to the control areas (Fig. 2*A*) and a positive mean effect size of removal (Fig. 2*B*) in all five study areas. All study areas included a positive effect of removals on apparent survival in the top model or in closely competing models (*SI Appendix*, Tables S2 and S3). The estimated mean increase in survival attributable to barred owl removal (mean effect size ± SE) ranged from a low of 0.044 ± 0.031 in Hoopa-Willow Creek (HUP-WC) to a high of 0.172 ± 0.077 in Cle Elum (CLE) (Fig. 2*B*). Estimates of mean effect size in CLE, COA, and HUP-WC indicated similar increases in survival to that observed in Klamath-Union/Myrtle (KLA-UM), but with greater uncertainty as shown by larger 95% confidence intervals (CI) that marginally bounded zero. The estimated effect of removals was consistently positive in all five study areas, which provided additional evidence of treatment effects beyond that provided by model selection results and 95% confidence intervals alone.

Movement probability of resident spotted owls from historical territories on control areas to territories on treatment areas (ψ_CT_) increased considerably in response to barred owl removal on two of the five study areas (CLE and COA) (Fig. 2*C* and *SI Appendix*, Table S4). In COA, estimates of ψ_CT_ increased from 0.012 ± 0.003 before removals to 0.094 ± 0.040 after removals (an 87% increase in movement; $\hat{\beta }$_*CTpost-removal*_ = 2.00 ± 0.56, 95% CI: 0.89, 3.10). Elsewhere, movement probability ranged from a low of 0.002 ± 0.001 in KLA-UM to a high of 0.024 ± 0.008 in CLE (Fig. 2*C*). Movement models that allowed ψ_CT_, ψ_TC_ to differ in CLE and GDR were competitive (*SI Appendix*, Table S4), but 95% CIs for estimated effect sizes overlapped zero in these study areas. We found weak evidence for sex-dependent effects on movement probabilities (*SI Appendix*, Table S4).

### Meta-analysis of Survival, Recruitment, and Population Change.

**Fig. 3.**
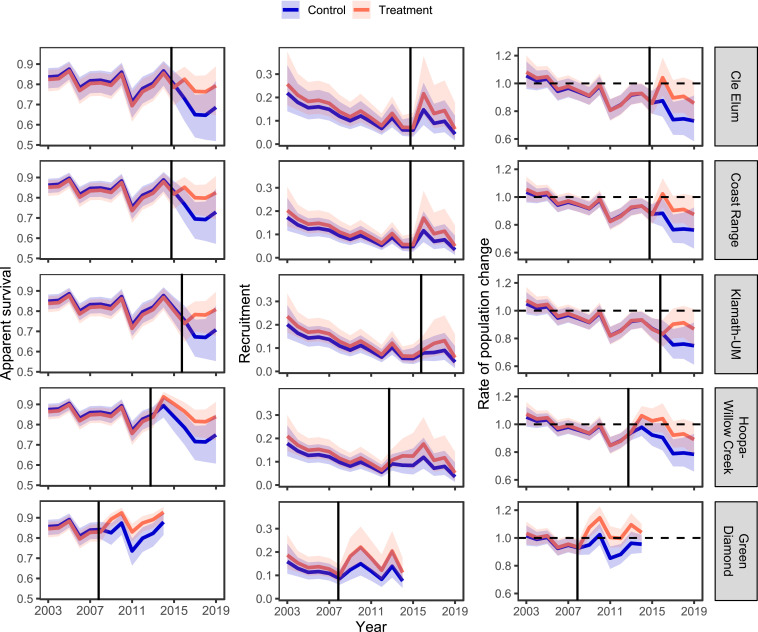
Meta-analysis of estimated effects of barred owl removal on long-term population dynamics of northern spotted owls in five study areas in Washington, Oregon, and California. We show annual estimates of apparent survival, recruitment, and the rate of population change for treatment (barred owls removed) and control (no removal) areas. Estimates are from the best meta-analysis model with all study areas combined. Shaded regions represent 95% CIs; solid vertical lines indicate the start-date of barred owl removal on treatment areas.

**Fig. 4.**
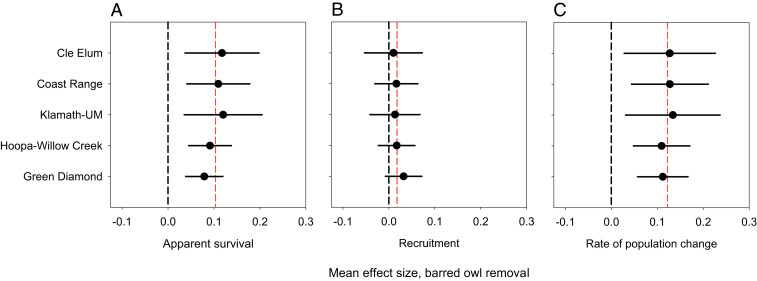
Mean change in vital rates and population trends of northern spotted owls that was attributable to barred owl removal based on a meta-analysis of five experimental study areas in Washington, Oregon, and California. We show estimates of mean effect size for (*A*) apparent survival, (*B*) recruitment, and (*C*) rate of population change of northern spotted owls. Mean effect size was calculated using Eq. 2 and weighted geometric means of before–after, control–treatment estimates from the best meta-analysis model. Red-dashed vertical lines indicate mean effect sizes across the five study areas. Positive values with a 95% CI that did not overlap zero (black-dashed vertical line) indicate strong evidence that removals increased a given vital rate in the treatment (barred owl removal) area relative to the control area.

We used a reparameterized temporal symmetry mark–recapture model (31, 32) with 6,661 captures and recaptures of 1,484 nonjuvenile spotted owls across all five study areas in a meta-analysis of apparent survival, recruitment, and annual rate of population change of spotted owls (*SI Appendix*, *Supplementary Text*). The best base model prior to testing for an effect of barred owl removal included additive effects of study area, treatment area, and year for both survival (φ) and recruitment (*f*), with individual random effects (σ_*p*_), year, and an interaction between study area and treatment area for capture probabilities (*p*). The addition of a BACI effect of barred owl removal to the best base model resulted in a new minimum Akaike’s Information Criterion corrected for small sample size (AIC_*c*_) model that contained 77% of the Akaike weight and was >22 times more likely (*SI Appendix*, Table S5). The top model indicated that barred owl removal had a strong, positive effect on apparent survival, and a positive, but weaker, effect on recruitment across all study areas (Fig. 3 and *SI Appendix*, Table S6). After removals, mean estimates of apparent survival on treatment areas (±SE) increased by 0.08 ± 0.02 (GDR) to 0.12 ± 0.04 (KLA-UM) relative to estimates on control areas (Figs. 3 and 4*A*); the overall mean increase in survival across study areas was 10%.

The effect of removals on recruitment was positive in the top model, but there was uncertainty in the magnitude of the increase as shown by 95% CIs of BACI regression coefficients (*SI Appendix*, Table S6), and mean effect sizes that included zero (Fig. 4*B*). A second model that received less support (ΔAIC_*c*_ = 2.46) included a strong, positive before–after effect of removal in treatment areas on both apparent survival ($\hat{\beta }$ = 0.61 ± 0.14, 95% CI: 0.34, 0.87) and recruitment ($\hat{\beta }$ = 0.55 ± 0.13, 95% CI: 0.29, 0.82). This second-ranked model received 3.4 times more support compared to the base model without removal effects, and together the top two models with removal effects contained 99.9% of the total AIC_*c*_ weight (*SI Appendix*, Table S5). Estimates of apparent survival were similar between these two models but estimates of recruitment from the top-ranked model indicated a concurrent increase on control areas during the removal period (Fig. 3) that was unaccounted for in the second-ranked model that lacked a before–after effect in control areas. Thus, we relied on estimates of survival and recruitment from the top-ranked model for final inferences and to derive estimates of the annual rate of population change (λ).

Before removing barred owls, mean estimates of annual population change ($\bar{\lambda }$_*t*_) on both treatment 0.953 (95% CI: 0.914 to 0.993) and control areas 0.948 (95% CI: 0.913, 0.984) indicated a general decline across all study areas, with an increasing annual rate of decline prior to removals (Fig. 3). After removing barred owls, estimates of $\bar{\lambda }$_*t*_ on treatment areas increased to 0.998 (95% CI: 0.899, 1.100), whereas estimates of $\bar{\lambda }$_*t*_ on control areas decreased to 0.879 (95% CI: 0.776, 0.983). These estimates correspond to postremoval declines of 0.2% and 12.1% per year for treatment and control areas, respectively. The mean increase in λ_*t*_ attributable to barred owl removals was positive with 95% confidence limits that excluded zero in all study areas (Fig. 4*C*). The mean increase in λ_*t*_ in treatment areas relative to control areas across all study areas combined was 0.114 (95% CI: –0.039, 0.267). With the exception of GDR, point estimates of λ_*t*_ in treatment areas were <1 during the final 3 y of the study (2017 to 2019), but the magnitude of annual declines was uncertain relative to that in control areas, as shown by upper 95% confidence limits that included 1.0 (stationary population) in areas with barred owl removal (Fig. 3).

## Discussion

Our long-term removal experiment provided a clear demonstration of the demographic consequences of emergent competition, and competitive release, between two previously allopatric predators. Removal of invasive barred owls had a strong, positive effect on survival of native spotted owls, which in turn alleviated long-term population declines of this federally threatened species. Barred owl removal had a positive, but weaker, effect on recruitment of spotted owls. The weaker effect of removals on recruitment was likely a consequence of consistently depressed reproduction of spotted owls (and diminished availability of new recruits) during the later years of the study (10, 11, 27). Removal of competitors also influenced the dispersal dynamics of resident spotted owls in at least two study areas (COA, CLE), as shown by a marked increase in the estimated probability of movement from territories in control areas to territories on treatment areas after barred owl occupants were removed.

The BACI design of the experiment was spatially replicated across much of the extent of the northern spotted owl’s geographic range in the United States, thereby providing strong inference across a variety of environmental conditions in terms of forest types, prey availability, and densities of spotted owls and barred owls. There was unlikely to be any systematic, sustained bias in factors affecting the results because of the long duration of the study, the extent of spatial replication, and similarities in forest (and disturbance) conditions between paired treatment and control sites (*SI Appendix*, Fig. S2). The results establish that the most substantial changes in population dynamics of northern spotted owls during the study were associated with the invasion, population expansion, and subsequent experimental removal of barred owls. We conclude that barred owl removal can aid in sustaining populations of spotted owls, but that outcomes may vary depending on the size of remnant populations of spotted owls, densities of established populations of barred owls, and magnitude of change in other stressors affecting spotted owls (e.g., habitat loss, climate).

### Demographic Consequences of Competitive Release.

The availability and distribution of old forests promotes survival (16) and territory occupancy (10, 17, 24) of both spotted owls and barred owls. As such, a limited supply of old-forest resources underlies much of the competitive relationship between the two owl species. As barred owls invaded the geographic range of northern spotted owls, they displaced and subsequently excluded spotted owls from their breeding territories via interspecific territoriality (16). Barred owls also exhibit a high degree of ecological overlap with spotted owls in terms of space use, habitat selection, and diets (33). This combination of interference and exploitation competition led to decreased survival and recruitment of territorial spotted owls, thereby exacerbating historical population declines associated with habitat loss. Once barred owls were removed, spotted owl survival, rate of population change, and to a lesser extent, recruitment, increased. The mechanism by which vital rates increased was that of competitive release (34), which occurred as the realized niche of spotted owls (compressed by competition from barred owls) (16, 35) approached the species’ fundamental niche with reduced competition. With barred owls removed, the recently restricted ecological niche of spotted owls had expanded back into the niche space subjugated by the invasive competitor.

Our analyses of individual study areas provided strong evidence that barred owl removal increased survival of spotted owls in two study areas (KLA-UM, GDR) with weaker, but biologically relevant, evidence of increases in survival in the remaining three. The strong effect of removals on survival in KLA-UM was somewhat unexpected because this study area had the least amount of barred owl removal effort (3 y) relative to the other four study areas (4 to 6 y). Subtle differences in the response of spotted owls to barred owl removal among individual study areas may reflect regional differences in: 1) numbers of remaining spotted owls, 2) density of territorial pairs of barred owls in treatment areas prior to removals, and 3) postremoval recolonization rates of barred owls from landscapes surrounding treatment areas (23, 27). In the CLE and COA study areas, for example, we attributed a relatively weaker effect of removals to the sparse number of marked spotted owls remaining by the end of the study (10 to 20 individuals), which limited our ability to detect a statistically precise effect of removals in these areas. In HUP-WC, preremoval differences in apparent survival between control and treatment areas appeared to reduce the magnitude (and precision) of a full BACI effect in this paired study area despite an average 3% increase in mean apparent survival of spotted owls on the treatment area following removals. The size and precision of the estimated demographic response of spotted owls to barred owl removal may have varied among individual study areas, but the resulting trends in treated areas were a consistent improvement over alternative trends documented in control areas without removal.

The limitations we encountered in detecting a strong effect of barred owl removal for some individual study areas were overcome in the meta-analysis, which used the combined power of all study areas as experimental replicates to estimate treatment effects. This analysis provided robust evidence that removals increased apparent survival of spotted owls by ∼10% across all study areas. The meta-analysis also indicated that the demographic impact of barred owl removal on spotted owls was similar across study areas, despite differences among study areas in the occurrence and recolonization rate of barred owls after removals (23, 27). Constancy in removal effort among study areas, especially in the vicinity of sites occupied by spotted owls, may have contributed to a similar removal effect on spotted owls across areas. We also had high confidence that barred owl colonists were quickly detected and removed throughout the year, regardless of variation among study areas in the level of recolonization. Barred owl colonists in treatment areas had little time to establish and defend territories.

Previous studies suggest that barred owls may disproportionately impact apparent survival relative to other demographic traits of spotted owls, which could represent either increased mortality or permanent emigration beyond study area boundaries (10–12). Our study not only provided experimental evidence to support these findings, but also a deeper understanding of the mechanisms by which barred owls affected survival and dispersal movements of spotted owls. In our multistate analysis of survival and movement, for example, we showed that spotted owls in the COA and CLE study areas that had been displaced by barred owls from their territories on control areas before removals were able to detect and settle on new territory openings in treatment areas after barred owls were removed. These findings support the hypothesis that competitive release from barred owls increased apparent survival of spotted owls in some cases by allowing displaced, nonterritorial spotted owls to regain a territory after the barred owl occupants had been removed (16, 23, 36). Our findings further demonstrated that territorial interactions with barred owls are a primary cause of increased breeding dispersal movements observed in spotted owls over the past three decades (36).

In northern spotted owls, reproduction promotes future recruitment of new individuals into the territorial component of the population, while the additive effects of recruitment and apparent survival of territory holders define λ_*t*_ (11). Reproductive output by spotted owls was low and variable during the later years of our study (11, 27), which may in part explain the lack of a strong effect of barred owl removal on spotted owl recruitment. Low reproduction during the removal period indicated there were few younger, nonterritorial recruits available in landscapes to fill territory vacancies once barred owl occupants were removed. Following removals, a general pattern across study areas was the maintenance of survival (and estimates of λ_*t*_) on treatment areas, with concurrent sharp decreases on control areas. This finding, coupled with low and variable recruitment, indicated that the immediate increase in λ_*t*_ on treatment areas relative to controls was a result of barred owl removal stabilizing apparent survival of resident spotted owls. With the exception of GDR, the estimated increase in survival (about 10%) was insufficient to result in positive population growth rates (i.e., λ_*t*_ > 1) near the end of the study period. Collectively, these results indicate that further increases in the annual rate of population change of spotted owls, or even maintaining stable rates of population change over time, will require increases in both reproductive rates and, subsequently, recruitment. Without additional recruitment, recovery and long-term persistence of spotted owls is unlikely.

Field experiments on the demographic consequences of competitive release between sympatric terrestrial predators are scarce. Notable exceptions include a field experiment on the impacts of reducing Eurasian badger (*Meles meles*) populations on densities of sympatric red foxes (*Velpes vulpes*) (37). Results demonstrated that culling badgers, which are considered the dominant and more aggressive species, substantially increased fox densities through a combination of interference and exploitation competition. Elsewhere, coyote (*Canis latrans*) removals have triggered increases in the abundance of sympatric mesopredators, including badgers, bobcats (*Felis rufus*), and gray foxes (*Urocyon cineroargenteus*) (38). More recently, large-scale BACI experiments in southern British Columbia showed that reducing rapidly expanding populations of moose (*Alces alces*) stabilized declining population trends of Woodland caribou (*Rangifer tarandus caribou*) by reducing abundance of wolves (*Canis lupus*), a shared predator (39). Similar to the results of our experiment, the moose-removal experiment showed how a single management action (species removal) can effectively halt population declines of focal species, but that actions addressing multiple limiting factors (e.g., habitat, climate) are required to achieve population growth and long-term persistence.

### Prospects for Management.

The barred owl removal experiment represents a culmination of sequential studies implemented in the same system over time, where demographic monitoring was first used to accumulate knowledge on the complexity of factors affecting population dynamics of spotted owls. Those observations were then formally tested within a large-scale experimental framework. The natural next step would be adoption of this evolving information state into a management context (40). Experimental results indicate that barred owl control can achieve rapid results in benefitting the persistence of northern spotted owls, at least over the short term. This does not suggest that barred owl control alone is sufficient to achieve recovery of spotted owls, as the availability of older forests is a necessary condition for barred owl removal to succeed. The rate of decline of spotted owl populations in control areas by the end of the study was severe (∼12% per year), indicating an increasingly high risk of these populations to local extirpations. A number of mechanisms that negatively affect small populations, including environmental stochasticity and Allee effects (11, 41), will make it increasingly difficult to recover spotted owl populations in some regions. Fast-moving development and implementation of management actions for barred owls based on experimental results, coupled with long-term management of suitable forest conditions, will be essential to the recovery and persistence of northern spotted owls.

The conservation and restoration of old forests, which has been a chief focus of recovery strategies for the northern spotted owl (19), is a major source of socio-economic controversy in the Pacific Northwest (42). The barred owl invasion has exacerbated this issue, placing an even higher ecological premium on remaining old conifer forests. Barred owls have become widespread and hyperabundant throughout much of the northern spotted owl’s geographic range (27). Even if barred owls can be maintained at low levels in some areas, we believe it is inevitable that the species will continue to exert substantial ecological pressure on spotted owls and other native wildlife. Broad-scale management of barred owls, including lethal removal, would require a long-term resource commitment, as any lapse in management could allow barred owls to quickly recolonize and erode conservation gains. This prospect raises questions about how long removals could and should be perpetuated; public acceptance and values associated with such actions are an important consideration (43).

Culling overabundant invasive species to manage their ecological impacts on target species is widely practiced, but outcomes are often unpredictable (43). Our study represents a promising example of successful removal and suppression of an invasive and increasingly abundant competitor, with a positive demographic response from a threatened native species. While suppression of barred owls can be difficult, costly, and ethically challenging, improvements in vital rates and population trends of spotted owls, and perhaps other threatened wildlife, can be expected when densities of barred owls are reduced from current levels. Alien predators are considered to be more harmful to prey populations than native predators (44), and the dynamic interactions between invasive and native predators can lead to profound changes in ecosystems by precipitating trophic cascades, often with considerable conservation and economic impacts (45, 46). In this sense, long-term management of barred owls may be critical not only to the preservation of spotted owls, but also to conservation of biodiversity in old-forest ecosystems of the Pacific Northwest.

## Materials and Methods

### Study Areas and Experimental Design.

The barred owl removal experiment was spatially replicated on five study areas distributed across the geographic range of the northern spotted owl (Fig. 1*D* and Table 1). All study areas had long-term, mark–recapture demographic data on northern spotted owls (10–12) and represented a range of different forest conditions cooccupied by spotted owls and barred owls (20). We included data from the pilot removal study in California (GDR), which included 1 additional year of barred owl removals and spotted owl demographic data not previously analyzed (23) (Table 1). The five study areas varied in climate, vegetation composition, and topography, but all were dominated by conifer or mixed conifer-hardwood forests (10, 20). The fieldwork occurred on federal, private, tribal, and state lands so that results and inferences would not be limited to certain ownerships and forest conditions.

Ideally in ecological experiments, treatment and control plots should be randomly selected and alternated during the study period to avoid the potentially confounding effects of unknown plot differences on results (47, 48). This was not possible in our study due in part to scale and logistics, but also because of considerations such as availability of pretreatment demographic data on spotted owls, land ownership restrictions, and the need to remove barred owls in the same areas over several years to limit compensatory immigration from surrounding landscapes (23, 27). Therefore, we divided each study area into two or more similar treatment (barred owls removed) and control (barred owls not removed) areas with respect to number of historical spotted owl territories, forest structural conditions within owl sites, and forest disturbance (e.g., wildfire, timber harvesting) (*SI Appendix*, S*upplementary Text*). This process resulted in five paired before–after treatment and control areas totaling 3,145 km^2^ and 3,404 km^2^, respectively (Fig. 1*D* and Table 1).

### Demographic Monitoring and Barred Owl Removal.

The removal experiment was conducted within the framework used to assess range-wide population status and trends of the northern spotted owl (10–12, 25, 49). As such, we integrated our experimental design and analysis into existing protocols used by these previous studies. Although demographic monitoring of spotted owls generally began in 1985 to 1990, we used 2002 as a common start year for demographic analyses across all study areas. This narrowed the pretreatment timeline of the experiment to a period when barred owls had become well established and were having measurable impacts on spotted owls (10). Spotted owls were surveyed during the breeding season of each year (March to August) using standardized protocols to document occupancy status of territories, locate and confirm previously banded owls, band unmarked owls, and document reproduction (50, 51).

We used barred owl-specific surveys to locate and remove barred owls throughout the year (26, 27). Our protocol for removals prohibited collection of nesting barred owls with dependent young, so removals were completed primarily in the nonbreeding season (September to April), or limited to barred owls not provisioning young during the breeding season. Barred owls detected in treatment areas were removed using a 12-gauge shotgun (22, 27). We did regular follow-up visits to detect colonizing owls and conduct additional removals as needed throughout the year. Repeated surveys and removals of barred owls indicated frequent and regionally variable recolonization of treatment areas by barred owls (23, 27). The single-visit detection rate of barred owls during surveys was generally high (66 to 74%) (26, 27), so we were confident that newly colonizing barred owls in treatment areas were quickly detected and removed. This dynamic of seasonally intermittent and temporary use of treatment areas by colonizing barred owls, which was a consistent pattern across experimental study areas, was in stark contrast to control areas where the majority of historical spotted owl territories were occupied by well-established resident pairs of barred owls (27). Removal and scientific collection of barred owls was approved by the Institutional Animal Care and Use Committee at Oregon State University and completed under Federal Fish and Wildlife and State Scientific Collection Permitting.

### BACI Analysis.

We determined the influence of barred owl removal on population dynamics of spotted owls using a paired BACI experimental design with long-term mark–recapture data. This approach permitted the impact of removal to be reliably distinguished from background time effects or underlying pretreatment differences in spotted owl vital rates between treatment and control areas. We conducted two different BACI analyses, where the first examined the effect of removals on survival and dispersal of spotted owls (i.e., movement between control and treatment sites) for each study area separately, and the second combined data from all five study areas in a meta-analysis of apparent survival, recruitment, and annual rate of population change (*SI Appendix*, S*upplementary Text*). For both analyses, we used program MARK to develop candidate models and estimate model parameters (52).

Previous studies of spotted owl population dynamics (10–12) and a pilot barred owl removal study (23) guided our analytical approach. We included all banded, territorial birds, and combined second-year and adult birds into a single age class (10, 11). For each analysis we began with a general fixed-effects model structure and then constrained model parameters in sequential sets (53), where capture probabilities (*p*) were modeled first, and the best structure was retained as we moved on to model focal demographic parameters. At each modeling stage, we used AIC_*c*_ and Akaike weights (*w*_*i*_) to select between competing models, while retaining the nonfocal parameters in their most general form. We generally selected the model with the lowest AIC_*c*_ value and highest Akaike weight (*w*_*i*_) as our best-supported model, but models within two AIC_*c*_ units (ΔAIC_*c*_ ≤ 2.0) were further evaluated as potentially competitive models (54).

Before testing for an effect of barred owl removal, we first investigated underlying variation in capture probabilities and vital rates of spotted owls with respect to: 1) treatment versus control areas, 2) time (categorical effects of year, and a continuous time trend [T]), 3) owl sex, and 4) study areas (meta-analysis only). For capture rates, we included models with an individual-specific random effect intercept term, σ_*p*_(.), to account for potential unexplained heterogeneity in capture rates among marked individuals (55) (*SI Appendix*, S*upplementary Text*). We assumed minimal overdispersion of the data (i.e., $\hat{c}$ = 1) because: 1) previous analyses of spotted owls detected little to no lack of independence of the data (12, 25), and 2) models with an individual random effect on capture probability are robust to overdispersion (55).

We tested for an effect of barred owl removal on vital rates of spotted owls by introducing a time (before–after) × treatment (control–impact) interaction to the best models characterizing baseline variation in sex, time, and preremoval differences between treatment and control areas. We specified a basic fixed-effects BACI model as:

$$\theta_{\mathrm{ij}}\;=\;\beta_{0}\;+\;\beta_{1}\;\left({\mathrm{period}}_{i}\right)\;+\;\beta_{2}\;\left({\mathrm{treated}}_{j}\right)\;+\;\beta_{3}\;\left({\mathrm{period}}_{i}\;\times \;{\mathrm{treated}}_{j}\right),$$ [1]

where *θ*_*ij*_ was a given vital rate between year *i* and (*i* + 1) on area *j*, *period*_*i*_ was a before-after indicator (0 before removals began and 1 after removal began), and *treated*_*j*_ was an indicator for treatment sites with barred owl removal (0 for areas never treated and 1 for areas treated at some point during the study). This model structure embraced classic BACI concepts in that it provided a direct test of whether changes in vital rates from the preremoval to postremoval time periods were different in treatment compared to control areas (23, 56, 57). Specifically, if removal of barred owls on the treated area had no effect on a given vital rate, the BACI interaction term (*β*_*3*_ in the example above) would be 0 because *β*_*3*_ measures pre- and posttreatment differences between treated and control areas. Thus, if *β*_*3*_ was >0, we concluded that removal of barred owls had a positive effect. In other words, *β*_*3*_ > 0 indicated that barred owl removal increased the vital rate above that expected by the pretreatment difference between controls and treatments. A positive *β*_*3*_ term with a 95% CI that did not overlap 0 was the strongest evidence of an effect. Positive effects with ≤10% of the 95% CI “slightly” overlapping 0 were regarded as weaker, but biologically relevant, evidence of an effect. We compared models with (*period × treated*) to an additive model (*period + treated*) and used evidence ratios to characterize the weight of evidence for models with barred owl removal effects (54). We also included a model with a before–after covariate (*BA:treat*) that was specific to treated sites during the removal period (56). Similar to a full BACI model, a model with *BA:treat* tested for a before–after change in vital rates on treated areas relative to control areas. A key difference between the two models was that the full BACI model allowed parameters to vary before and after removals on both treatment and control areas, whereas a model with *BA;treat* (and two fewer parameters) allowed vital rates to vary before and after removals on treatment areas only (i.e., assumes no before–after change on control areas).

We estimated the mean amount of change in vital rates that could be attributed to barred owl removal (mean effect size) as:

$$\left({\hat{\bar{\theta }}}_{treatment:after}\;-\;{\hat{\bar{\theta }}}_{control:after}\right)\;-\;\left({\hat{\bar{\theta }}}_{treatment:before}\;-\;{\hat{\bar{\theta }}}_{control:before}\right),$$ [2]

where $\hat{\bar{\theta }}$ was the weighted geometric mean of annual estimates for a given vital rate from the best BACI model. We used the reciprocal of variances and the variance-covariance matrix output from MARK to calculate weighted means, SEs, and 95% CIs. Positive values with a 95% CI that did not overlap zero provided evidence that removals increased a given vital rate on treated areas relative to controls.

## Supplementary Material

Supplementary File

## Data Availability

Data included in the manuscript and supporting information are available to qualified researchers upon request from the corresponding author.
