# Supplementary material for: Invader removal triggers competitive release in a threatened avian predator
Source: Proc Natl Acad Sci U S A. 2021 Jul 19;118(31):e2102859118. doi: 10.1073/pnas.2102859118 (PMC8346899; doi:10.1073/pnas.2102859118)
Supplement: Supplementary File [file pnas.2102859118.sapp.pdf]

Supplementary Information for

Invader removal triggers competitive release in a threatened avian predator

J. David Wiens[a,*], Katie M. Dugger[b], J. Mark Higley[c], Damon B. Lesmeister[d], Alan B. Franklin[e], Keith A. Hamm[f], Gary C. White[g], Krista E. Dilione[a], David C. Simon[a], Robin R. Bown[h], Peter C. Carlson[g], Charles B. Yackulic[i], James D. Nichols[j], James E. Hines[j], Raymond J. Davis[d], David W. Lamphear[f], Christopher McCafferty[d], Trent L. McDonald[k], Stan G. Sovern[l].

[a]U.S. Geological Survey, Forest and Rangeland Ecosystem Science Center, Corvallis, Oregon, USA. [b]U.S. Geological Survey, Oregon Cooperative Fish and Wildlife Research Unit, Department of Fisheries and Wildlife, Oregon State University, Corvallis, Oregon, USA. [c]Hoopa Tribal Forestry, Hoopa, California, USA. [d]USDA Forest Service, Pacific Northwest Research Station, Corvallis, Oregon, USA. [e]U.S. Department of Agriculture, Wildlife Services, National Wildlife Research Center, Fort Collins, Colorado, USA. [f]Green Diamond Resource Company, Korbel, California, USA. [g]Department of Fish, Wildlife, and Conservation Biology, Colorado State University, Fort Collins, CO 80523 USA. [h]U.S. Fish and Wildlife Service, Oregon State Office, Portland, Oregon, USA. [i]U.S. Geological Survey, Southwest Biological Science Center, Flagstaff, Arizona, USA. [j]U.S. Geological Survey, Patuxent Wildlife Research Center, Laurel, Maryland, USA. [k]McDonald Data Sciences, LLC, Laramie, Wyoming, USA. [l]Department of Fisheries and Wildlife, Oregon State University, Corvallis, Oregon, USA.

Corresponding author: J. David Wiens
Email: jwiens@usgs.gov

This PDF file includes:

Supplementary text
Figures S1 to S2
Tables S1 to S6
SI References

Appendix S1. *Mark-recapture analyses of the effect of barred owl removal on population dynamics of northern spotted owls.*

Analysis of survival and movement on individual study areas. We used a multistate survival model (1–3) to estimate state-specific apparent survival (*S*) and recapture probability (*p*), in addition to state transition probabilities (ψ), where we defined states as areas with (treatment) or without (control) barred owl removal. This modeling framework facilitated before-after control-treatment (BACI) comparisons of spotted owl survival while simultaneously accounting for dispersal movements (state transitions) by spotted owls between territories located in treatment and control areas. Dispersal between territories with and without barred owl removal might be expected if resident spotted owls that were displaced from their historical territories by barred owls in control areas had detected and settled on newly vacant territories in treated areas after removals. For each annual sampling occasion, we defined two states that reflected whether an individual spotted owl's annual activity center was located on the control (state C) or treatment area (state T). Thus, there were two state-transition probabilities of interest: ψ_{CT}, the probability of movement from control to treatment sites between years, and ψ_{TC}, the probability of movement from treatment to control sites between years. A detected individual's state was determined each year using the single best location to represent the owl's territory center based on nest locations, presence of young, or repeated observations during the breeding season.

We tested for an effect of barred owl removal on survival of spotted owls using the BACI approach outlined above, where an effect of barred owl removal was added to the best baseline model that did not include a removal effect. For movement, our primary interest was in examining the research hypothesis that dispersal from control to treatment areas increased in response to barred owl removal. We examined this hypothesis by specifying a model that allowed estimates of ψ_{CT} during the removal period to vary from estimates of ψ_{TC} or pre-removal estimates of ψ_{CT}, (model {TC, $CT_{post\text{-}removal}$}). Using AIC_c we compared this model to four alternative models assuming: 1) equivalent movement probability between treatment and control areas (model {TC=CT}); 2) state-specific movement probabilities (model {TC, CT}); 3) sex-dependent movement probabilities (model {sex}); or 4) additive effects of state-specific movement and sex (model {TC, CT + sex}). We detected no movements by spotted owls between control and treatment areas at HUP-WC during the study period, so we fixed movement parameters at zero for this area. There were no movement events detected in KLA-UM during the removal period, so we excluded model {TC, $CT_{post\text{-}removal}$} from the candidate set of models for this study area.

Meta-analysis of survival, recruitment, and population rate of change. We conducted the meta-analysis of barred owl removal effects on apparent survival (φ), recruitment (*f*), and annual rate of population change (λ) using the reparametrized likelihood of the temporal symmetry models (4, 5) implemented in program MARK. We used the survival and recruitment parameterization of the model, where estimates of λ_t were derived as $\hat{\varphi}_t + \hat{f}_t$ (4, 5). Here, $\hat{\varphi}_t$ reflected both survival and retention of territory holders in the study area, and $\hat{f}_t$, the number of new animals in the population at time *t* + 1 per animal in the population at time *t*, reflected both in situ recruitment and immigration of new recruits from outside the study area (2). Previous meta-analyses of northern spotted owls used a random effects approach to examine trends in vital rates of northern spotted owls, where independent study areas were considered the sampling units (6–8). This approach suggested a global model with a group by time interaction as the basis for random effects modeling of demographic parameters. This was not practical in our study, in part because of sample size limitations near the end of the study period, but also because the initial stages of our modeling procedure supported additive treatment and time (year) effects, rather than their interaction, which would not have been an appropriate basis for random effects models. We therefore relied on a fixed-effects approach to modeling demographic parameters. We did, however, examine support of individual-specific random effects on capture rates (σ_p) as in the multistate analysis of individual study areas.

We handled movements of spotted owls between treatment and control areas (coded as group effects in MARK) by truncating a bird's capture history at the last year it was observed in one area group, and then beginning a new capture history the year it was detected in the new area group. The start year and duration of barred owl removals varied by study area (Table 1),

which was accommodated using the design matrix in MARK. Data from GDR were not available for 2015 – 2019, so we treated these years as missing observations and fixed associated model parameters to zero. Initial effects considered for parameters in the Link-Barker model were general time, sex, and group (control-treatment) effects. As in the multistate analysis of individual study areas, we used AIC_c to sequentially fit and compare models with and without an effect of barred owl removal and calculated mean effect size using the model containing the BACI contrast.

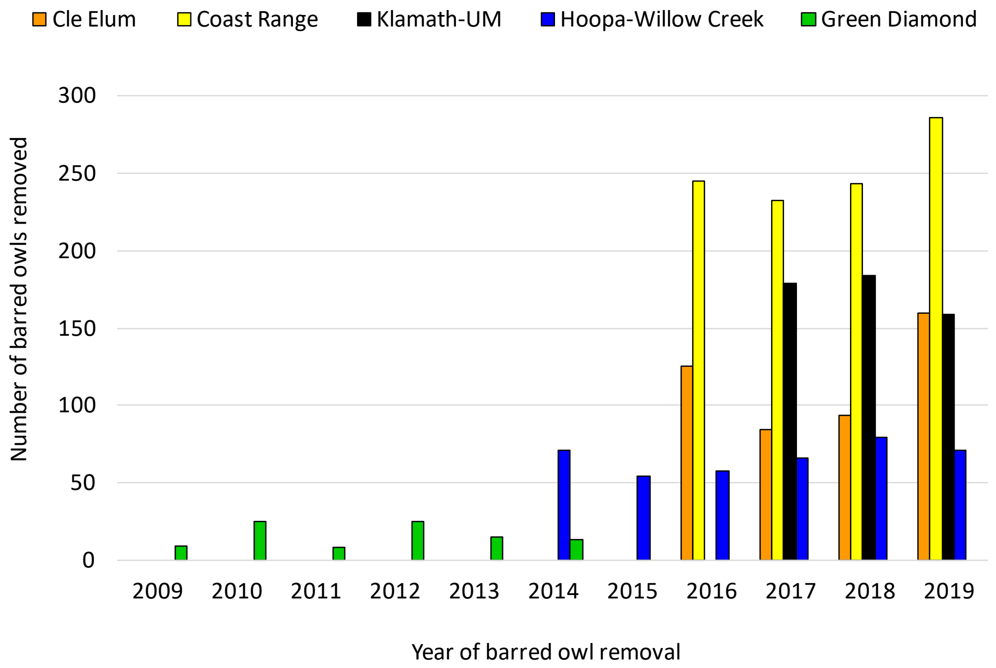

Figure S1. Annual numbers of barred owls removed from treatment segments of five study areas used to estimate the effect of barred owl removal on population dynamics of northern spotted owls. Removal of barred owls was approved by the Institutional Animal Care and Use Committee at Oregon State University and completed under Federal Fish and Wildlife and State Scientific Collection Permitting.

Appendix S2. *Assessment of forest condition and disturbance on experimental study areas.*

Population dynamics of spotted owls are tied to the availability and distribution of older forest structural conditions within breeding territories (6–8). Although not a goal of our study, we assessed possible differences in habitat suitability or forest disturbances (e.g., wildfire, timber harvest) between areas with (treatment) and without (control) barred owl removal. This was relevant because selection of control and treatment sites was not fully randomized across study areas, so we checked for possible treatment-specific changes in forest conditions, or disturbances, that may have coincided with the barred owl removal period. Climate conditions also affect population dynamics of northern spotted owl (9). We reasoned, however, that regional climate conditions would influence spotted owls similarly on paired treatment and control areas of each study area, such that it was unlikely for climate effects to be confounded with the effects of barred owl removal.

We measured forest and disturbance conditions using methods outlined in other studies of spotted owl population dynamics (7, 8, 10; Table S1). We first merged the boundaries of individual territories historically used by spotted owls (Theissen polygons; 7, 8) within treatment and control areas of each study area separately. Next, we calculated the annual proportion of habitat (or disturbance) within the total combined area of each merged polygon. Measures of forest conditions were specific to year, study area, and control versus treatment sites. There were no substantial changes in forest conditions or disturbance during the removal period in the five study areas (Fig. S2).

Table S1. Metrics used to characterize forest conditions on areas with and without barred owl removal in five experimental study areas in Washington, Oregon, and California.

**Forest conditions**	**Description**
Proportion of area with disturbance (DIST)	Proportion of moderate to high severity forest disturbance in each study area during the 3-yr interval prior to each survey year (7, 8, 10).
Proportion of area with forest edges (EDGE)	Proportion of nesting/roosting cover type that occurs along edges of nesting/roosting cover patches or is contiguous with a patch and interfused with other cover types (7, 8, 10)
Proportion of area with suitable habitat (HAB)	Proportion of the nesting and roosting cover type used by northern spotted owls (7, 8, 10)

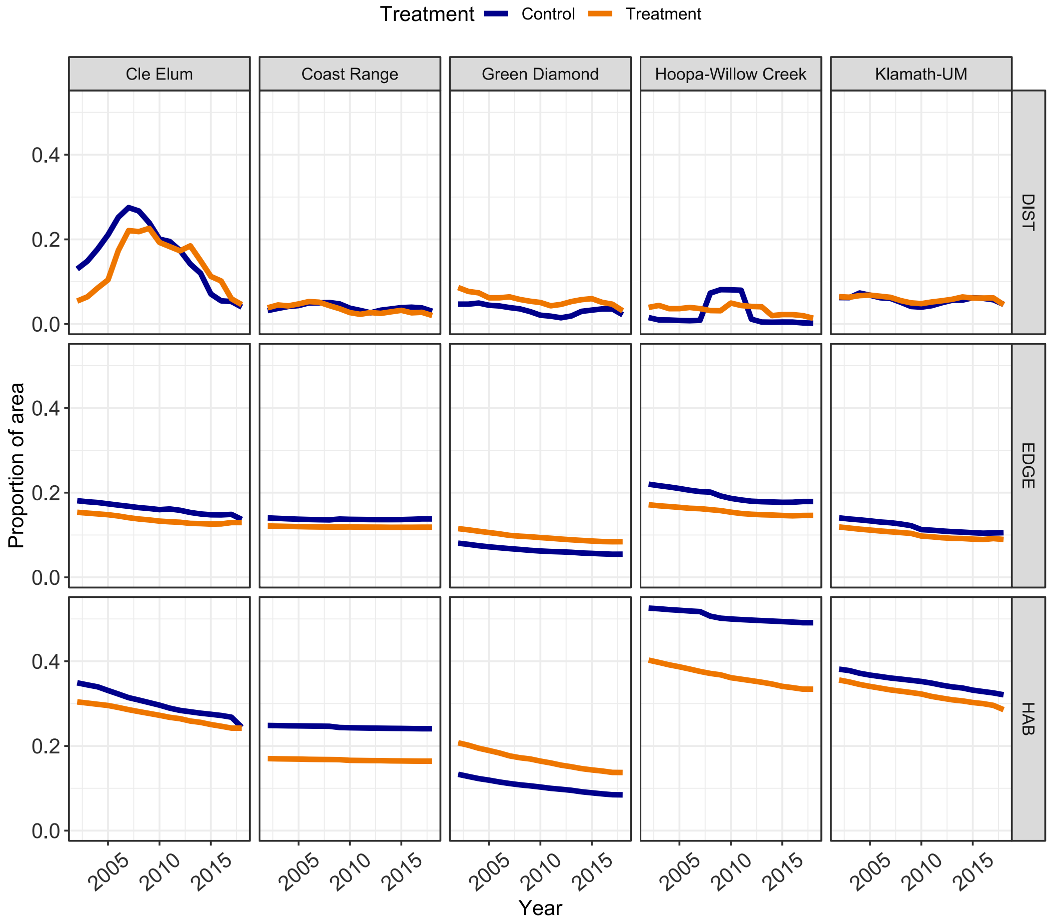

Figure S2. Year- and area-specific values of forest habitat conditions on areas with (treatment) and without (control) barred owl removal in five different study areas used to estimate the effect of barred owl removal on population dynamics of northern spotted owls, 2002 – 2018 (estimates not available for 2019). We show annual estimates of the proportion of area with habitat disturbance (DIST), forest edges (EDGE), and suitable spotted owl habitat (HAB).

Appendix S3. *Individual heterogeneity in spotted owl capture probabilities.*

Analyses of the effect of barred owl removal on spotted owls included candidate models for recapture rates (*p*) that included an individual-specific random effect intercept term, $\sigma_p(.)$, used to account for potential unexplained heterogeneity in recapture rates among marked individuals (3, 11). If the ratio of the AIC$_c$ weight of the model with $\sigma_p(.)$ divided by the weight of the model without $\sigma_p(.)$ was >10, we considered the weight of evidence for individual heterogeneity in recapture rates to be strong and retained $\sigma_p(.)$ when modeling demographic parameters of spotted owls (8). Below, we provide modeling results relative to $\sigma_p(.)$ for each mark-recapture analysis used to determine the effect of barred owl removal on vital rates of spotted owls.

Analysis of survival and movement on individual study areas. The best initial fixed-effects model for capture rates were constant (no time, treatment, or sex effects) for one study area (CLE), or had the effect of sex (COA; higher for males), treatment level (KLA-UM; higher on treatment area), or the additive effects of sex and treatment level (GDR; higher for males on treatment areas). Multistate models that accounted for individual heterogeneity in recapture probabilities greatly outperformed traditional models that did not (Table S2) and estimates of variance (σ_p) and associated 95% confidence intervals were > 0 for all study areas. Consequently, we retained $\sigma_p(.)$ in all models used to evaluate focal demographic parameters. Annual estimates of capture probabilities (i.e. means of the individual heterogeneity distributions) in the final top models for each study area were generally high, ranging from a mean low of 0.760 (SE = 0.061) for females in COA to a mean high of 0.943 (SE = 0.011) for both sexes in HUP-WC.

Meta-analysis of survival, recruitment, and population rate of change. Similar to the multistate analyses of individual study areas, we found strong evidence of individual heterogeneity in the meta-analysis of recapture rates for spotted owls (Table S5). Estimated variance of individual random effects in the top model was 1.76 (SE = 0.12, 95% CI = 1.52 to 2.03). Thus, we retained $\sigma_p(.)$ in all models used to evaluate focal demographic parameters. Annual estimates of capture probabilities (i.e. means of the individual heterogeneity distributions) in the final top model ranged from a low of 0.69 (SE = 0.082) to a high of 0.996 (SE = 0.003).

Table S2. Ranking of candidate multistate mark-recapture models used to test for an effect of barred owl removal on apparent survival of northern spotted owls in each of five study areas in Washington, Oregon, and California.

Study area	Multistate apparent survival (*S*)[a]	ΔAIC_c	w_i	*K*	Deviance
Cle Elum	BA:treated	0.00	0.52	5	578.16
	Intercept only (.)	1.78	0.22	4	581.99
	period + treated + period × treated	2.47	0.15	7	576.50
	period + treated + period × treated + T_{pre}	4.27	0.06	8	576.22
	period + treated	4.76	0.05	6	580.86
	year × treated (full model)	36.97	0.00	36	546.14
	year × treated (full model; *without* σ_p)	65.33	0.00	35	574.49
Coast Range	year	0.00	0.52	22	1225.31
	BA:treated + year	0.95	0.33	23	1224.16
	period + treated + year	3.98	0.07	24	1225.08
	period + treated + period × treated + year	4.19	0.06	25	1223.19
	year × treated (full model)	7.56	0.01	37	1200.92
	period + treated + period × treated + T_{pre}	15.51	0.00	10	1265.65
	Intercept only (.)	19.82	0.00	6	1278.11
	year × treated (full model; *without* σ_p)	141.64	0.00	36	1335.00
Klamath-UM	T	0.00	0.29	6	1445.12
	BA:treated + T	0.06	0.28	7	1443.16
	period + treated + period × treated + T_{pre}	0.35	0.25	9	1439.43
	period + treated + period × treated	1.52	0.14	8	1442.61
	period + treated	4.90	0.03	7	1448.00
	Intercept only (.)	5.82	0.02	5	1452.94
	year × treated (full model)	28.97	0.00	70	1341.61
	year × treated (full model; *without* σ_p)	99.63	0.00	69	1418.65
Hoopa-Willow Creek	Intercept only (.)	0.00	0.28	3	1058.17

	treated	0.53	0.22	4	1056.70
	treated + BA:treated	1.29	0.15	5	1055.44
	BA:treated	1.65	0.12	4	1057.81
	period + treated	2.19	0.10	5	1056.34
	period + treated + period × treated + T_{pre}	2.77	0.07	7	1052.88
	period + treated + period × treated	3.20	0.06	6	1055.33
	year × treated (full model)	19.82	0.00	36	1010.20
	year × treated (full model; *without* σ_p)	61.40	0.00	35	1055.97
Green Diamond	treated + BA:treated	0.00	0.57	8	1226.15
	period + treated + period × treated	1.85	0.22	9	1225.98
	period + treated + period × treated + T_{pre}	2.77	0.14	10	1224.89
	period + treated	5.07	0.04	8	1231.22
	treated	7.98	0.01	7	1236.15
	Intercept only (.)	8.10	0.01	6	1238.28
	year × treated (full model)	10.48	0.00	29	1193.87
	year × treated (full model; *without* σ_p)	108.71	0.00	28	1294.16

[a]Models include a before-after indicator for all time intervals after barred owl removal (period), a control-impact indicator for treated areas with barred owl removal (treated), a continuous time-trend (T), a continuous time-trend before barred owl removal (T_{pre}), and a before-after indicator that was specific to treated areas during the removal period (BA:treated). We compared models with a *period* × *treated* term to models without this term to evaluate support for an effect of barred owl removal. Models with a *BA:treated* covariate tested for a before-after change in apparent survival on treatment areas only. All models included individual random effects on capture rates (σ_p) unless noted otherwise. We include the intercept only (.), full model, and full model without σ_p for comparisons. ΔAIC_c: difference in AIC_c compared to the model with the lowest AIC_c value; w_i: Akaike weight; *K*: number of parameters in the model.

Table S3. Best multistate mark-recapture models containing an effect of barred owl removal on apparent survival (*S*) of northern spotted owls in each of five study areas in Washington, Oregon, and California. We include coefficients ($\hat{\beta}$), standard errors (SE), and lower (LCL) and upper (UCL) 95% confidence limits for the effects of: 1) a before-after control-impact change in survival between areas with (treated) and without (control) barred owl removal (period × treated); and 2) a before-after difference on treated areas (BA:treated).

Study area	Best model[a]	Effect	ΔAIC_c	$\hat{\beta}$	SE	LCL	UCL
Cle Elum	*S*(period + treated + period × treated)	period × treated	2.47	3.38	6.05	−8.48	15.25
	S(BA:treated)	BA:treated	0.00	3.23	8.15	−12.73	19.20
Coast Range	*S*(period + treated + period × treated + year)	period × treated	4.19	0.79	0.59	−0.36	1.95
	S(BA:treated + year)	BA:treated	0.95	0.57	0.54	−0.50	1.63
Klamath-UM	*S*(period + treated + period × treated + T*pre*)	period × treated	0.35	0.87	0.38	0.11	1.62
	S(BA:treated + T)	BA:treated	0.06	0.42	0.32	−0.19	1.04
Hoopa-Willow Creek	*S*(period + treated + period × treated + T*pre*)	period × treated	2.77	0.34	0.34	−0.32	1.01
	S(BA:treated + treated)	BA:treated	1.29	0.26	0.24	−0.20	0.73
Green Diamond	*S*(period + treated + period × treated)	period × treated	1.85	0.59	0.26	0.09	1.09
	S(BA:treated + treated)	BA:treated	0.00	0.51	0.16	0.19	0.83

[a]Models include a before-after indicator for all time intervals after barred owl removal (period), a control-impact indicator for treated areas with barred owl removal (treated), a continuous time-trend (T), a continuous time-trend before the removal period (T_{pre}), and a before-after indicator that was specific to treated areas after removals (BA:treated).

Table S4. Ranking of candidate multistate mark-recapture models used to test for an effect of barred owl removal on movement probability of northern spotted owls between territories with (treatment) and without (control) barred owl removal in five study areas in Washington, Oregon, and California.

Study area	Movement probability model[a]	ΔAIC_c	w_i	K	Deviance
Cle Elum	TC=CT	0.00	0.39	36	574.49
	TC, $CT_{post\text{-}removal}$	0.41	0.31	37	572.48
	*sex	1.99	0.14	37	574.06
	*TC, CT	2.42	0.12	37	574.49
	TC, CT + sex	4.42	0.04	38	574.06
Coast Range	TC, $CT_{post\text{-}removal}$	0.00	0.96	52	1286.17
	TC=CT	7.92	0.02	51	1296.32
	sex	8.58	0.01	52	1294.75
	TC, CT	10.14	0.01	52	1296.31
	TC, CT + sex	10.79	0.00	53	1294.72
Klamath-UM	TC=CT	0.00	0.45	52	1436.40
	sex*	2.10	0.16	53	1436.40
	TC, CT*	2.10	0.16	53	1436.40
	TC, CT + sex*	4.19	0.06	54	1436.40
Green Diamond	TC=CT	0.00	0.40	28	1294.16
	TC, CT	1.11	0.23	29	1293.21
	sex*	1.96	0.15	29	1294.06
	TC, $CT_{post\text{-}removal*}$	2.05	0.14	29	1294.16
	TC, CT + sex*	3.03	0.09	30	1293.08

[a]Models include: equivalent movement probability between treatment and control areas (TC=CT), state-specific movement probabilities (TC, CT), movement probabilities depended on owl sex only (sex), additive effects of state-specific movement and sex (TC, CT + sex), or estimates of ψ_{CT} during the removal period vary from estimates of ψ_{TC} or pre-removal estimates of ψ_{CT}, (TC, $CT_{post\text{-}removal}$). Model (TC, $CT_{post\text{-}removal}$) was not estimable for the Klamath-UM study area (i.e. no movement during the post-removal period). ΔAIC_c: difference in AIC_c compared to the model with the lowest AIC_c value; w_i: Akaike weight; K: number of parameters in the models.

*Models include uninformative parameters (i.e. 95% confidence limits of regression coefficients widely overlapped zero, and similar value of deviance to top model).

Table S5. Ranking of candidate mark-recapture models used in the meta-analysis of the effect of barred owl removal on apparent survival (φ), recruitment (*f*), and annual rate of population change (λ) of northern spotted owls across five study areas in Washington, Oregon, and California.

Model	ΔAIC_c	w_i	K	Deviance
φ(period × treated + area + year) σ_p(.) *p*(area × treated + year) *f*(period × treated + area + year)	0.00	0.77	76	5109.07
φ(BA:treated + area + year) σ_p(.) *p*(area × treated + year) *f*(BA:treated + area + year)	2.46	0.23	74	5115.62
φ(period + treated + area + year) σ_p(.) *p*(area × treated + year) *f*(period + treated + area + year)[a]	15.42	0.00	74	5128.59
φ(treated + area + year) σ_p(.) *p*(area × treated + year) *f*(treated + area + year)	43.32	0.00	72	5160.58
φ(sex + treated + area + year) σ_p(.) *p*(area × treated + year) *f*(sex + treated + area + year)	46.09	0.00	74	5159.25
φ(area × treated + year) σ_p(.) *p*(area × treated + year) *f*(area × treated + year)	48.25	0.00	80	5149.13
φ(area + year) σ_p(.) *p*(area × treated + year) *f*(area + year)	52.45	0.00	70	5173.79
φ(treated + year) σ_p(.) *p*(area × treated + year) *f*(treated + year)	56.41	0.00	64	5190.00
φ(treated + area + year) σ_p(.) *p*(treated + area + year) *f*(treated + area + year)	60.60	0.00	68	5186.03
φ(area + treated × year) σ_p(.) *p*(area × treated + year) *f*(area + treated × year)	61.45	0.00	104	5112.97
φ(area × treated + year) σ_p(.) *p*(area × treated) *f*(area × treated + year)	67.29	0.00	63	5202.92
φ(treated × year) σ_p(.) *p*(area × treated + year) *f*(treated × year)	74.67	0.00	96	5142.68
φ(area × treated) σ_p(.) *p*(area × treated + year) *f*(area × treated)	79.31	0.00	48	5245.45
φ(area × treated) σ_p(.) *p*(area × treated) *f*(area × treated)	84.37	0.00	31	5284.92
φ(area × treated + sex) σ_p(.) *p*(area × treated) *f*(area × treated + sex)	88.33	0.00	34	5282.83
φ(treated + area + year) σ_p(.) *p*(area × treated × year) *f*(treated + area + year)	98.37	0.00	221	4903.98
φ(area × treated + year) σ_p(.) *p*(area × treated) *f*(area × treated × year)	99.87	0.00	168	5017.98

φ(area × treated × year) σ_p(.) *p*(area × treated + year) *f*(treated + area × year)	104.68	0.00	200	4955.09
φ(area × treated × year) σ_p(.) *p*(area × treated + year) *f*(area × treated + year)	106.37	0.00	207	4941.88
φ(area × treated × year) σ_p(.) *p*(area × treated) *f*(area × treated + year)	128.66	0.00	189	5002.41
φ(area × treated × year) σ_p(.) *p*(area × treated) *f*(area × treated × year)	174.61	0.00	293	4824.40
φ(area × treated + year) *p*(area × treated + sex + year) *f*(area × treated + year)[b]	389.90	0.00	80	5490.78

[a]Base model without effects of barred owl removal.
[b]Best model without individual random effects on recapture rates (sigma-p). ΔAIC_c: difference in AIC_c compared to the model with the lowest AIC_c value; w_i: Akaike weight; *K*: number of parameters in the model.

Table S6. Coefficients ($\hat{\boldsymbol{\beta}}$), standard errors (SE), and lower (LCL) and upper (UCL) 95% confidence limits from the best mark-recapture model of apparent survival (φ) and recruitment (*f*) in the meta-analysis of the effect of barred owl removal on population dynamics of northern spotted owls across five study areas in Washington, Oregon, and California.

**Model parameter**[a]	$\hat{\boldsymbol{\beta}}$	**SE**	**LCL**	**UCL**
Survival (φ)				
Intercept	0.978	0.402	0.189	1.766
period	-0.037	0.171	-0.372	0.298
treated	-0.084	0.072	-0.225	0.057
period × treated	0.645	0.164	0.324	0.966
area (COA)	0.047	0.121	-0.190	0.284
area (CLE)	-0.158	0.158	-0.467	0.151
area (KLA-UM)	-0.056	0.111	-0.274	0.162
area (HUP-WCSA)	0.151	0.110	-0.064	0.366
Recruitment (*f*)				
Intercept	-3.993	0.592	-5.153	-2.833
period	0.535	0.204	0.136	0.934
treated	0.162	0.079	0.008	0.317
period × treated	0.225	0.178	-0.124	0.574
area (COA)	0.083	0.130	-0.173	0.338
area (CLE)	0.320	0.166	-0.005	0.644
area (KLA-UM)	0.233	0.117	0.003	0.463
area (HUP-WCSA)	0.114	0.114	-0.108	0.337

[a]We used a logit link function to model survival parameters, and a log-link function to model recruitment.

SI References

1. J. D. Nichols, W. L. Kendall, The use of multi-state capture-recapture models to address questions in evolutionary ecology. *J. Appl. Stat.* 22, 835–846 (1995).
2. J. D. Lebreton, R. Pradel, Multistate recapture models: modelling incomplete individual histories. *J. Appl. Stat.* 29, 353–369 (2002).
3. G. C. White, W. L. Kendall, R. J. Barker, Multistate survival models and their extensions in Program MARK. *J. Wildl. Manage.* 70, 1521–1529 (2006).
4. W. A. Link, R. J. Barker, Modeling association among demographic parameters in analysis of open population capture–recapture data. Biometrics 61, 46–54 (2005).
5. R. Pradel, Utilization of capture-mark-recapture for the study of recruitment and population growth rate. *Biometrics* 703–709 (1996).
6. E. D. Forsman *et al.*, Population demography of northern spotted owls. *Studies in Avian Biology* 40, 1–97 (2011).
7. K. M. Dugger *et al*., The effects of habitat, climate, and barred owls on long-term demography of northern spotted owls. *Condor* 118, 57–116 (2016).
8. A. B. Franklin *et al*., Range-wide declines of northern spotted owl populations in the Pacific Northwest: a meta-analysis. *Biol. Conserv.* (In press).
9. E. M. Glenn, R. G. Anthony, E. D. Forsman, Population trends in northern spotted owls: associations with climate in the Pacific Northwest. *Biol. Conserv.* 143, 2543-2552 (2010).
10. R. J. Davis, *et al*., "Northwest Forest Plan–the first 20 years (1994-2013): status and trends of late-successional and old-growth forests." Gen. Tech. Rep. PNW-GTR-911. Portland, OR: US Department of Agriculture, Forest Service, Pacific Northwest Research Station. 112 p. (2015).
11. O. Gimenez, R. Choquet, Individual heterogeneity in studies on marked animals using numerical integration: capture–recapture mixed models. *Ecology* 91, 951–957 (2010).
